# Artificial Intelligence in Eye Disease: Recent Developments, Applications, and Surveys

**DOI:** 10.3390/diagnostics12081927

**Published:** 2022-08-10

**Authors:** Jae-Ho Han

**Affiliations:** 1 Department of Brain and Cognitive Engineering, Korea University, 145 Anam Rd., Seoul 02841, Korea; hanjaeho@korea.ac.kr; 2 Department of Artificial Intelligence, Korea University, 145 Anam Rd., Seoul 02841, Korea

## 1. Introduction

Artificial intelligence (AI) has expanded by finding applications in medical diagnosis for clinical support systems [1]. The use of AI in ophthalmology is drawing enormous interest in diagnosing various ophthalmic diseases that have traditionally been delicate and/or thought to be difficult to precisely diagnose by clinical experts [2]. In particular, AI can assist ophthalmologists in accurate diagnosis by integrating recently developed technologies when applied to funduscopy scans, optical coherence tomography (OCT), and visual field examination to achieve powerful classification performance in detecting corneal and retinal anomalies [3]. For example, AI can be used in miscellaneous ocular images as a plausible solution for screening, diagnosing, and monitoring patients with major eye illnesses in the anterior and posterior segments of primary care [4]. 

In addition, with the aid of deep learning (DL) methods in ophthalmic images, various disorders can be inspected by observing retinal scans to efficiently detect macular and choroidal abnormalities, bleeding, vessel defects, and glaucoma [5]. In other words, DL architectures are used for learning to recognize a variety of eye-related diseases in ophthalmology to improve diagnosis rates with clinically acceptable performance, compared to ophthalmology specialists [6]. Thus, AI could effectively serve as a reliable safety platform for both patients and doctors, and as an auxiliary tool to promptly judge the results; this could not only reduce the possibility of misdiagnosis, but could also improve patient experience by expediting efficient treatment [7]. 

Furthermore, many automated eye-related disease screening and analysis medical devices have also been successfully applied in clinical practice as the hardware instrumentation and measurement tools, which can be combined with AI algorithms [8]. Besides OCT, ophthalmic diagnostics devices can be subdivided into refractors, corneal topography systems (machines), retinal ultrasound systems, tonometer, etc. [9]. For instance, vision screening can be performed by using photoscreeners and autorefractors—the former enables us to identify the risk for developing amblyopia, such as media opacity, ocular alignment and ptosis, and the latter can detect risk factors and the eye conditions that may cause decreased vision and amblyopia [10]. Hence, with the aid of the precise medical devices and automated instruments, AI could reduce the traditional inefficiency or barriers and increase efficacy and safety for ophthalmology once fully implemented, in conjunction with conventional diagnostic and therapeutic methods and protocols.

The aims of the Special Issue are to highlight the above-mentioned trends in the field by exploring recent developments, applications, and research reviews (https://www.mdpi.com/journal/diagnostics/special_issues/AI_Eye). AI methods, such as machine learning and deep learning, have shown feasibility for screening, detecting, diagnosing, and monitoring common eye diseases, not only in diverse clinical practice, but also in basic ophthalmology research. With a total of 11 research articles, the Special Issue comprises studies of eye diseases on anterior segments of the ocular structure, such as the cornea, and posterior segments, such as the retina.

Among these studies, Sánchez-Morales et al. presented a *K*-nearest neighbor (*K*-NN) algorithm-based ensemble model that can improve the capabilities of diagnosing glaucoma [11]. The authors demonstrated a novel soft voting method that integrates the probability of class membership gained by three different machine learning models, where the proposed ensemble model was able to enhance the capability of diagnosis.

For the early detection of corneal ulcers, Alquran et al. suggested a support vector machine (SVM) method for classifying corneal images to automatically extract features for discriminating ulcers from a general grading perspective [12]. Consequently, they achieved more than 90% accuracy based on a public dataset, where colored features were extracted from three color spaces, namely, red-green-blue (RGB), luminance chroma-blue chroma-red (YCbCr), and hue-saturation-value (HSV).

Kim et al. proposed an automatic DL algorithm to identify the optic disc and cup to estimate the risk of glaucoma [13]. This study introduced a multiscale average pooling network (MAPNet) and mask region-based convolutional neural network (Mask R-CNN) for segmenting the boundaries of the aforementioned region of interest (ROI) and accordingly, estimating key features, such as the dice coefficient (DC) and neuroretinal rim-to-disc (RD) ratio from retinal fundus images.

In the study by He et al., timely diagnosis of age-related macular degeneration (AMD) was realized from OCT images using the local outlier factor (LOF) algorithm and DL method [14]. A ResNet-50 model with L2-constrained softmax loss was retrained for feature extraction from the available dataset images, where the proposed LOF algorithm functioned as the classifier. The detection accuracies were compared over different datasets to determine the efficiency of the proposed approach in detecting AMD.

Alryalat et al. demonstrated a modified U-net DL model for predicting the response to intravitreal anti-vascular endothelial growth factor (anti-VEGFs) injections in patients with diabetic macular edema (DME) [15]. The results, based on the treatment-response analyzer system using OCT images, show that the segmentation and classification accuracies are comparable to those of ophthalmology residents and retina specialists.

A support tool for the segmentation of retinal functional layers in cross-sectional images was explored by employing graph theory and geodesic distance [16]. The proposed work takes advantage of interleaving various gradients, such as horizontal, vertical, or open-closed gradients, for diverse conditions of hundreds of OCT B-scan datasets. Consequently, the average values of the mean absolute error and signed error were confined to a few couple of pixels.

A deep learning algorithm was also tested for screening diabetic retinopathy (DR) by applying CNNs to the specific employed database [17]. The authors found that their method performed well in detecting any DR and classifying eyes with referable DR (RDR) for retinographies of type 2 diabetes mellitus (DM) cases, in terms of accuracy (ACC), sensitivity (S), specificity (SP), positive predictive value (PPV), and negative predictive value (NPV).

The authors of [18] were able to diagnose bacterial keratitis (BK) and fungal keratitis (FK) in a retrospective study of slit-lamp images based on their DL model. Specifically, CNN segmentation and classification models were developed to distinguish both types of keratitis. In comparison to corneal specialists, the models outperformed in terms of identification accuracies, which were significantly improved for both keratitis diagnoses.

An AI-based screening tool was established for the automatic assessment of pterygium by extracting infected regions from those frequently exposed to sunlight radiation [19]. In this study, the authors integrated a spatial pyramid pooling module (PPM) and group convolution into the DL segmentation network. They evaluated the best variant model that exhibited segmentation performances for the mean accuracy, mean intersection over union, Hausdorff distance, and Jaccard index (JI).

Furthermore, two review papers intensively surveyed the AI methods in eye diseases [20,21]. The first scrutinized a computer-assisted system for early screening of pterygium based on DL networks that were able to successfully classify pterygium tissue images, localize the lesion tissues, and semantically segment them [20]. Secondly, Jeong et al. surveyed recent studies on DL-based automated screening and diagnosis of retinal diseases, such as DR, AMD, and glaucoma, where the challenges in developing these systems were also discussed [21].

In summary, the papers published in the Special Issue demonstrate and disseminate the recently developed cutting-edge AI methods for automated detecting systems of various anterior and posterior segments of ocular diseases, which can also provide insights and challenges to researchers, practitioners, and clinical experts in ophthalmology. Finally, the guest editor would like to express his sincere gratitude to all the authors who contributed to the Issue, all the anonymous referees who voluntarily devoted themselves to reviewing the manuscripts, and the wonderful editorial staff, with a special compliment to Mr. Edwin You for the dedicated support.
